# Staging Accuracy and Prognostic Value of Prostate-Specific Membrane Antigen PET/CT Strongly Depends on Lymph Node Tumor Burden

**DOI:** 10.3390/jcm13216534

**Published:** 2024-10-30

**Authors:** Oktay Özman, Hans Veerman, Roberto Contieri, Matteo Droghetti, Maarten L. Donswijk, Marinus J. Hagens, Pim J. Van Leeuwen, André N. Vis, Henk G. van der Poel

**Affiliations:** 1 Department of Urology, The Netherlands Cancer Institute, 1066 CX Amsterdam, The Netherlands; h.veerman@nki.nl (H.V.); m.hagens@nki.nl (M.J.H.); pj.v.leeuwen@nki.nl (P.J.V.L.); a.vis@amsterdamumc.nl (A.N.V.); h.vd.poel@nki.nl (H.G.v.d.P.); 2 Prostate Cancer Network the Netherlands, 1066 CX Amsterdam, The Netherlands; 3 Department of Urology, Amsterdam University Medical Centers, 1066 CX Amsterdam, The Netherlands; 4 Department of Biomedical Science, Humanitas University, Via Rita Levi Montalcini 4, 20090 Milan, Italy; 5 Division of Urology, IRCCS Azienda Ospedaliero-Universitaria di Bologna, 40138 Bologna, Italy; droghet@gmail.com; 6 Department of Nuclear Medicine, The Netherlands Cancer Institute, 1066 CX Amsterdam, The Netherlands; m.donswijk@nki.nl

**Keywords:** prostate-specific membrane antigen, positron emission tomography, computed tomography, prostate cancer, prostatectomy, lymph node, staging

## Abstract

**Objectives**: To explore the factors affecting the lymph node metastasis (LNM) detection performance of prostate-specific membrane antigen positron emission tomography/computed tomography (PSMA PET/CT) and to evaluate its prognostic value for biochemical recurrence after radical prostatectomy (RP). **Methods**: Patients who had intermediate- or high-risk prostate cancer and underwent robot-assisted (RA)RP between 2017 and 2021 were included. Initial lymph node staging was carried out using PSMA PET/CT. Sensitivity, specificity, and positive (PPV) and negative (NPV) predictive values were calculated. A cut-off value for LNM tumor deposit size that maximizes specificity was investigated and a post hoc specificity analysis was carried out. In survival analysis for biochemical progression-free survival (bPFS) after RP, Kaplan–Meier curves of molecular imaging (mi)N0 and miN1 patients were compared using the log-rank test and separate Cox regression models were developed to reveal the significance of PSMA PET/CT staging in pre- and post-surgery settings. **Results**: In 583 patients with a prevalence of pathology-proven LNM of 27.4%, overall sensitivity, specificity, PPV, and NPV of PSMA PET/CT per patient were 26.3% [95%CI 18.9–35.5], 93.9% [95%CI 84.9–100], 61.8% [95%CI 44.5–83.5], and 77.1% [95%CI 69.7–85.1], respectively. PSMA PET/CT showed a better sensitivity as LNM tumor deposit size increased (*p* = 0.003 OR 2.4 [95%CI 1.3–4.4]) and a better specificity in pT3-4 tumors (96.1%) versus pT2 (91.1%, *p* = 0.024 OR 2.7 [95%CI 1.1–6.3]). After adjustment according to 5.5 mm LNM tumor deposit size, which showed the best discriminative performance (AUC: 0.905 [95%CI 0.804–1.000, *p* < 0.001]), overall sensitivity tripled (90.2%, *p* < 0.001). The 1-year bPFS was 56.0% and 83.3% for miN1 and miN0 patients, respectively (*p* < 0.001). Whereas miN0pN1 was not, miN1pN1 disease was independently associated with decreased bPFS (HR:2.1 95%CI 1.3–3.4, *p* < 0.001). **Conclusions**: PSMA PET/CT has a lymph node tumor burden-dependent and cohort-driven diagnostic ability but consequently a strong independent prognostic value for predicting biochemical recurrence after RARP.

## 1. Introduction

Lymph node metastasis (LNM) is a key phenomenon of prostate cancer (PCa) biology that triggers a pathway resulting in PCa-related mortality. Extended pelvic lymph node dissection (ePLND) stands as the gold standard lymph node staging modality for intermediate- and high-risk PCa [1], but still, the technique needs more guidance from pre- and peri-operative imaging modalities. Preoperative assessment of LNM has been traditionally carried out using abdominal computed tomography (CT) or magnetic resonance imaging (MRI), which assess lymph nodes in size and morphology [2]. However, these features are not reliable enough to detect malignant lymph nodes. ePLND decision still depends on clinical risk factors (initial prostate-specific antigen [PSA] level, Gleason score, tumor stage, and so forth) [3], and the dissection area is a preassumed template according to pelvic anatomical landmarks [4]. But contemporary robot-assisted surgery and integrated technologies/techniques allow surgeons to collect more and accurate lymph nodes from a patient-tailored dissection area.

Molecular imaging that targets the prostate-specific membrane antigen (PSMA) has been one of the game-changing advancements in the management of PCa [5]. PSMA positron emission tomography (PET)/CT has a superior discriminative ability to detect LNM compared to CT and MRI [6]. A systematic review and meta-analysis showed that PSMA PET/CT outperformed traditional imaging modalities with an >80% specificity rate [7]. Nevertheless, the sensitivity rate ranged between 33% and 97%. The factors related to the wide variation in the reported sensitivity of PSMA PET/CT for LNM have not been studied well yet. Moreover, beyond its diagnostic abilities, PSMA PET/CT has also a prognostic value which has been clearly demonstrated in recurrent or metastatic disease settings but has not been studied well in localized and locally advanced PCa. Therefore, the aim of this study was (1) to explore factors affecting the LNM detection performance of PSMA PET/CT using robot-assisted ePLND outcomes as a reference and (2) to evaluate its prognostic value for biochemical recurrence after RP.

## 2. Materials and Methods

### 2.1. Patient Selection

The current retrospective study was approved by the local institutional review board (IRBd22-051). Patients provided an informed consent form at the start of treatment. The data of patients who had biopsy-proven intermediate- or high-risk PCa according to the European Association of Urology classification between 2017 and 2021 in a single high-volume center were retrieved from the database. All patients were subjected to a preliminary comparison depicting patient selection for robot-assisted radical prostatectomy (RARP) and ePLND, which provided a reference (histopathological examination of lymph nodes) for the evaluation of PSMA PET/CT as a diagnostic test. Afterwards, those who underwent RARP and ePLND (study cohort) were subjected to further diagnostic analysis. Patients who had missing data, who underwent salvage RP, or who underwent treatment in a different center were excluded.

### 2.2. PSMA PET/CT Imaging

PSMA PET/CT imaging was performed using a Philips Gemini TF-II or Vereos (Philips Healthcare^®^, Amsterdam, The Netherlands/Orlando, FL, USA). 68Ga-Glu-urea-Lys (Ahx)-HBED-CC ([68Ga]Ga-PSMA-11) was radiolabeled in house using a fully automated system (Scintomics GmbH, Munich, Germany). Scanning commenced approximately 45 min post injection (PI) after a fixed dose of approximately 100 MBq intravenously combined with acquisition times of 3 min per bed position (min/bp) for the pelvis and 2 min/bp for the remainder of the scan range. From September 2019, the tracer dose was increased to a 150 MBq fixed dose combined with acquisition times of 4.0 min per bed position (min/bp) for the pelvis and 2.5 min/bp for the remainder of the scan range.

2-(3-{1-carboxy-5-[(6-[18F]fluoro-pyridine-3-carbonyl)-amino]-pentyl}-ureido)-pentanedioic acid ([18F]DCFPyL) purchased through BV Cyclotron (Amsterdam, the Netherlands) was administered as an intravenous bolus injection with a fixed dose of 200 MBq. Scanning commenced approximately 60 min PI, with 2 min/bp over the complete scan range. PET images were combined with a non-contrast-enhanced CT (120–140 kV and 40–80 mAs with dose modulation) for attenuation correction and anatomical reference. All PET images were corrected for scatter, decay, and random coincidences.

In 2017, PSMA PET/CT was performed in patients who had clinical T-stage (cT) > 3a, biopsy International Society of Urological Pathologists (ISUP) grade > 3, or PSA level > 20 ng/mL. In 2019, the criteria were expanded to patients who had D’Amico intermediate unfavorable-risk PCa and in 2020, PSMA PET/CT was also performed in intermediate favorable-risk PCa. All PSMA PET/CTs were assessed and reported by the same team of expert nuclear medicine specialists in line with E-PSMA guidelines [8] and discussed in multidisciplinary board meetings.

### 2.3. Robot-Assisted Extended Pelvic Lymph Node Dissection

Radical prostatectomy was performed in a robot-assisted laparoscopic approach using the da Vinci S(i) or Xi surgical robot (Intuitive Inc., Sunnyvale, CA, USA). Extended PLND was performed if the preoperative probability of LNM was >7% according to the Briganti Nomogram, as previously described [9]. Patients were diagnosed with LNM of PCa using the gold standard histopathological examination of ePLND samples. Pathological examination of the nodal material was performed as described previously [10].

### 2.4. Statistics

The primary outcomes were the accuracy of PSMA PET/CT for detecting regional pelvic LNM and biochemical progression-free survival (bPFS) after RP.

The number of true positives, false positives, true negatives, and false negatives was calculated based on imaging and histology outcomes. Sensitivity, specificity, and positive (PPV) and negative (NPV) predictive values were calculated per patient.

Two separate logistic regression models were developed to test for factors that might be associated with the sensitivity and specificity of PSMA PET/CT. These are as follows: PSA at diagnosis, age, pathological features of the tumor (pT stage [T2 vs. T3-4], ISUP grade groups), type of radiolabeled tracer ([68Ga]Ga-PSMA-11- or [18F]DCFPyL), and number of lymph nodes resected. LNM tumor deposit size and number of positive lymph nodes were the additional variables of the regression test for sensitivity.

To evaluate the performance of PSMA PET\CT for different LNM tumor deposit sizes, area under curve (AUC) values of receiver operating characteristic (ROC) curves (as means of the estimated sensitivity and specificity) were calculated in pN1 patients. A value of LNM size that maximizes the Youden’s index (sensitivity + specificity − 1) was considered as a cut-off [11]. A post hoc sensitivity analysis was carried out after adjustment of the pN1 disease definition according to the aforementioned cut-off value (positive lymph nodes with a tumor deposit smaller than the cut-off value were assumed to be below PET detection limits and excluded) [12].

Biochemical PFS was calculated as the time from RARP to biochemical (PSA) recurrence, which was defined as at least two consecutive PSA serum levels > 0.2 ng/mL at least 3 months after RARP, demonstrated using Kaplan–Meier curves, and compared between patients who have PSMA PET/CT-positive lymph nodes (miN1) and those who do not (miN0), using the log-rank test in the high-risk PCa subgroup. A Cox regression model was developed to reveal the significance of PSMA PET/CT staging in pre- (D’Amico criteria) and post-surgery (post-RP PSA [persistence vs. non-detectable PSA], pathological Gleason score and T stage (pT2 vs. pT3-4), surgical margin status [R0 vs. R1], and miN substratified pN stage [pN0 vs. miN0pN1 vs. miN1pN1]) settings for bPFS after RARP.

Continuous and categorical variables were displayed with medians with interquartile ranges (IQRs) and frequencies with percentages, respectively. Comparisons of continuous and categorical variables were carried out using Mann–Whitney U and chi square tests, respectively. All statistical analyses were performed using SPSS version 21.0 (IBM, Armonk, NY, USA) and MedCalc v 23.0.6. (MedCalc Software Ltd., Ostend, Belgium). A *p*-value < 0.05 was considered statistically significant.

## 3. Results

In total, 583 patients’ data were included in the diagnostic analyses (study cohort). To address the effect of patient selection for surgical treatment of PCa on the study cohort shaping (survivorship bias), we also report the features of the non-surgery cohort with a preliminary comparative analysis (Table 1).

### 3.1. Non-Surgery Cohort

Of 385 patients who were staged using PSMA PET/CT but had not undergone RARP + ePLND, 81 had distant metastasis (cM1) and the remaining received various treatments: definitive radiotherapy (n = 237), watchful waiting (n = 24), active surveillance (n = 10), inclusion in clinical trials (n = 6), hormonotherapy (n = 11), and other (n = 16). Patients in the non-surgery group were older and had higher initial PSA levels and more frequent clinically advanced local tumors (cT3-4) compared to the study group (all *p* < 0.001). miN1-staged patients more frequently underwent non-surgical treatments (30.3% vs. 11.7%, *p* < 0.001) (Table 1).

### 3.2. Study Cohort

The baseline characteristics of patients who underwent RARP + ePLND for D’Amico intermediate- to high-risk PCa are shown in Table 1. Of the 583 patients, 160 (27.4% [95%CI 23.3–32.0]) had regional lymph node metastasis (pN1). The median numbers of resected and positive lymph nodes were 18 [IQR: 13–24] and 1 [IQR: 1–2.3], with no difference in the number of lymph nodes resected between pN0 and pN1 patients (18 [IQR: 13–24], 18 [IQR: 14–23]; *p* = 0.67). In the study group, PSMA PET/CT was performed in 460 and 123 patients using 68Ga-PSMA and [18F]DCFPyL radioisotopes, respectively, and detected clinically suspect nodes (miN1) in 68 (11.7%) patients. Overall sensitivity, specificity, PPV, and NPV of PSMA PET/CT per patient were 26.3% [95%CI 18.9–35.5], 93.9% [95%CI 84.9–100], 61.8% [95%CI 44.5–83.5], and 77.1% [95%CI 69.7–85.1], respectively (Table 2). The accuracy and diagnostic odds ratio were 75.3 [95%CI 71.6–78.8] and 5.40 [95%CI 3.24–7.45], respectively.

In the logistic regression analysis, the sensitivity of PSMA PET/CT was only associated with the LNM tumor deposit size (*p* = 0.003 OR 2.4 [95%CI 1.3–4.4]) and the specificity of it was only associated with the pT stage of the primary tumor (*p* = 0.024 OR 2.7 [95%CI 1.1–6.3]). Specificity was significantly lower in patients with a prostate-confined tumor (pT2: 91.1%) compared to patients with a locally advanced primary tumor (pT3-4: 96.1%).

In 160 pN1 patients, the median diameter of intranodal metastasis was 3 [IQR: 2–6] mm, with significantly larger metastases in miN1 patients (miN0: 3 [IQR: 2–4.8]; miN1: 8 [IQR: 5.5–9.5] mm; *p* < 0.001). PSMA PET/CT showed a better performance as metastasis size increased (AUC:0.905 [95%CI 0.804–1.000, *p* < 0.001]). A threshold of 5.5 mm for metastasis size displayed the highest Youden’s index with ≥80% sensitivity and specificity values (80% and 84%, respectively). After adjustment according to the 5.5 mm LNM tumor deposit size, overall sensitivity tripled (90.2%, *p* < 0.001).

### 3.3. Survival Analysis

In the study cohort, 175 (30.0%) patients developed biochemical recurrence after RARP with a 1.6 [IQR: 0.9–2.5] year monthly follow-up. miN1 (39, 51.3%) patients had a significantly higher rate of recurrence compared to miN0 patients (136, 26.8%; *p* < 0.001). The 1-year bPFS was 56.0% and 83.3% for miN1 and miN0 patients, respectively (*p* < 0.001). miN1 disease was associated with decreased bPFS independently from both pre-surgery (HR:2.2 95%CI 1.5–3.2, *p* < 0.001) and post-surgery (HR:2.1 95%CI 1.3–3.4, *p* < 0.001) parameters, separately. Whereas miN0pN1 disease did not have an increased risk of recurrence, miN1pN1 patients had the same risk 2-fold increased (Table 3).

## 4. Discussion

In this contemporary cohort study of intermediate- to high-risk PCa patients treated with RARP and ePLND at a tertiary cancer center, the overall sensitivity and specificity of PSMA PET/CT for detecting regional pelvic LNM were 26.3% and 93.9%, respectively. A systematic review including the meta-analysis of 10 studies and 701 individuals’ data has shown that 68Ga-PSMA PET/CT has a sensitivity of 84% and specificity of 95% for primary lymph node staging of patients with intermediate- or high-risk PCa [7]. Across the studies, the specificity of 68Ga-PSMA PET/CT remained >80% steadily, except for a value of 67% in one of the earliest reports, whereas sensitivity varies within a wide range of 33–100%. The studies had a heterogeneous population of intermediate- and high-risk patients. The lowest sensitivity rate was reported by Yaxley et al. with 208 patients, which contained a comparable rate of intermediate-risk individuals to the present study [13]. We found a similar sensitivity rate of PSMA PET/CT with a similarly distributed cohort and pN1 disease prevalence. The relatively low sensitivity rate was explained by the high frequency of small metastases (median 3 mm) caused by resecting a median of 18 lymph nodes per ePLND session.

pN1 disease is an epiphenomenon of more aggressive and advanced PCa and therefore it is not easy to measure such tumor-related factors’ (i.e., ISUP score and T stage) effect on the sensitivity (miN1/pN1) of PSMA PET/CT. Nevertheless, it appeared to be strongly related to the lymph node metastasis burden. Contrary to morphological examination of lymph nodes with traditional radiological imaging methods, as a molecular imaging method, PET/CT provides a functional assessment of tumor cells. Thus, the intranodal metastasis burden becomes more important than the anatomical size of the lymph node invaded by the cancer. We found that PSMA PET/CT showed a sensitivity of 90% for LNMs > 5.5 mm. In Zhang’s study, which reported a 93% sensitivity rate with PSMA PET/CT, the median metastasis size was 13 mm [14]. In the current literature, there are diameter values for PSMA PET/CT-detected tumor deposits in LNMs, which do not markedly deviate from our findings. Budäus et al. and Hope et al. reported that the median sizes of PSMA PET/CT-detected LNM tumor deposits were 13.6 and 11.0 mm, respectively, which were significantly larger than the corresponding values of non-detected ones (4.3 and 6 mm, respectively) [15,16]. Similar values were revealed by another group of authors investigating LNM tumor deposit diameters associated with a PSMA PET/CT detection rate of 90% (longitudinal 6.3 mm and short 4.9 mm) [17].

Given the nature of the design of the studies, all reported papers on the accuracy of PSMA PET/CT suffer from a kind of patient selection bias that mostly occurs in survival analyses. In real-life clinical settings, some number of patients undergo non-surgical treatments that never provide pathological tissue confirmation of lymph node involvement. In our previous study, we found that patients who had miN1 disease were less likely to undergo RP in our center [18]. Consequently, our miN1 disease rate in the RP + ePLND cohort was relatively low (12%) compared to current data in the literature. Studies with miN1 disease rates comparable to those of our group showed poor sensitivity rates similar to ours. Yet, studies claiming higher sensitivity rates also reported a higher incidence of miN1 disease. This positive correlation between miN1 rate and sensitivity may be explained by the steadily high specificity of PSMA PET/CT for LNM. Thus, every miN1 case included in analysis is more likely to supply true positivity than false positivity. Eventually, PSMA-driven patient selection for ePLND results in a relatively high sensitivity rate. In our center, the Briganti Nomogram is still a key factor in ePLND decision-making, which makes the sensitivity rate of PSMA PET/CT less affected by patient selection bias in such a way [19].

Alongside this quantitative effect, patient selection also interferes with the performance of PSMA PET/CT in a qualitative way. Our recent findings regarding the association of LNM tumor deposits with the performance of PSMA PET/CT also showed that calculating the diagnostic characteristics of PSMA PET/CT by taking ePLND outcomes into account as reference weakens in centers where patients harboring a high LNM burden receive RT instead of RP. Clinical decision-making between RP and non-surgical treatments which lack tissue diagnosis determines the gap between the calculated and the real diagnostic performance of PSMA PET/CT.

It was clearly demonstrated that PSMA PET/CT cannot replace histopathological lymph node evaluation using ePLND specimens, due to the lymph node tumor burden-dependency of PSMA avidity [14,15,16]. For exactly the same reason, it may be a good candidate to be a prognostic factor beyond the diagnostic imaging method. In our cohort, PSMA PET/CT detected a certain amount of LNMs not randomly but highly sensitively (in LNM tumor deposits > 5 mm). Thus, PSMA PET/CT successfully stratified a high-risk PCa population. This means that this highest prognostic group consists of two separable subgroups behaving significantly differently in terms of biochemical recurrence after RP. Previously, we showed the prognostic value of PSMA positivity for shorter follow-ups and with adjustment for clinical parameters [9,19]. In this recent analysis, we found that the prognostic value of the PSMA positivity of lymph nodes was also operational in a post-surgery setting with pathological parameters. The significance of miN1 disease in a pre-surgery setting was clearly transferred to miN1pN1 disease in a post-surgery setting. Probably, a proper ePLND was enough to eliminate miN0pN1 metastases, which has no significant prognostic value in our analysis. While the prognostic value of PSMA was clearly depicted in this metastatic disease, data in localized and locally advanced disease in this regard are still lacking [20]. Our study provided unique findings which can shed light on the use of PSMA positivity both in primary and post-RP assessments. Changing the paradigm in the use of PSMA PET/CT in the primary assessment of newly diagnosed PCa can also meet the need for a fourth criteria of D’Amico, which allows a new risk group: very-high-risk PCa. In this highly selected subgroup of patients who are more likely to recur after RP + ePLND, treatment can be structured multimodally by taking adjuvant RT and concomitant androgen deprivation therapy into consideration while counseling patients before surgery.

Our study is not devoid of limitations mainly related to its retrospective design. In particular, our results are based on data from a tertiary cancer center (where interpretation of the PSMA PET/CT images was carried out by experienced readers and final clinical decisions were made in regular multidisciplinary team meetings) and may not be applicable to other centers. Also, our data were lacking for executing a lymph node-based evaluation of PSMA PET/CT, alongside per-patient analysis.

## 5. Conclusions

In conclusion, PSMA PET/CT has a lymph node tumor burden-dependent, cohort-driven diagnostic ability over ePLND but consequently a strong independent prognostic value for predicting biochemical recurrence after RARP. It rules out lymph metastases of locally advanced primary tumors (pT3-4) with excellent specificity; however, its calculated sensitivity weakens for patients who have a high LNM burden, who were referred to RT instead of RP + ePLND. PSMA PET/CT detects >5 mm lymph node tumor deposits much more sensitively.

## Figures and Tables

**Table 1:** The baseline characteristics of patients.

	Study Cohort	Non-Surgery Cohort	*p*-Value
Number of Patients	583	304	
Age at Diagnosis; Median (IQR)	68.0 (64.0–72.0)	70.0 (64.0–75.0)	<0.001
PSA (ng/mL); Median (IQR)	10.5 (7.0–20.0)	12.0 (8.3–25.0)	<0.001
Clinical Tumor Stage; N (%)			<0.001
cT1	104 (17.8)	55 (18.1)	
cT2	327 (56.0)	138 (45.4)	
cT3	151 (25.9)	101 (33.2)	
cT4	1 (0.3)	10 (3.3)	
Biopsy Gleason Score; N (%)			<0.001
6	20 (3.4)	30 (9.9)	
7	246 (42.2)	151 (49.6)	
8	173 (29.7)	57 (18.8)	
9–10	144 (24.7)	66 (21.7)	
Molecular Imaging Nodal Stage; N (%)			<0.001
miN0	515 (88.3)	212 (69.7)	
miN1	68 (11.7)	92 (30.3)	
Briganti Score (%); Median (IQR)	19.3 (11.3–39.6)	26.6 (10.5–65.4)	<0.001
Pathological Tumor Stage; N (%)			
pT0	1 (0.2)		
pT2	206 (35.3)		
pT3	376 (74.6)		
pT4	0 (0.0)		
Pathological Gleason Score; N (%)			
6	7 (1.2)		
7	377 (65.8)		
8	57 (10.0)		
9–10	132 (23.0)		
Pathological Nodal Stage; N (%)			
pN0	423 (72.6)		
pN1	160 (27.4)		

**Table 2:** Diagnostic performance of PSMA PET/CT in overall study cohort and subgroups of patients therein.

	True Positive	False Positive	True Negative	False Negative	Sensitivity	Specificity	Positive Predictive Value	Negative Predictive Value
Overall	42	26	397	118	26.3	93.9	61.8	77.1
After Adjustment for LNM Tumor Deposits > 5.5 mm	37	26	397	4	90.2	93.9	58.7	99.0
EAU Risk Stratification								
Intermediate-risk (n = 77)	5	4	57	11	31.3	93.4	55.6	83.8
High-risk (n = 506)	37	22	340	107	25.7	93.9	62.7	76.1
pT Stage								
pT2 (n = 207)	1	17	174	15	6.3	91.1	82.0	68.4
pT3-4 (n = 376)	41	9	223	103	28.5	96.1	5.6	92.1
Tracers								
^68^Ga (n = 460)	27	19	333	81	25.0	94.6	58.7	80.4
[^18^F]DCFPyL (n = 123)	15	7	64	37	28.9	90.1	68.2	63.4
Number of Lymph Nodes Resected								
1–10 (n = 75)	3	3	54	15	16.7	94.7	50.0	78.3
11–20 (n = 285)	22	10	192	61	26.5	95.0	68.8	75.9
21–30 (n = 169)	14	7	116	32	30.4	94.3	66.7	78.4
>30 (n = 54)	3	6	35	10	24.1	85.4	33.3	77.8

**Table 3:** Multivariable Cox regression models for biochemical progression-free survival.

Covariates	*p* Value	HR	95%CI (Lower–Upper)	
*Post-surgery setting*				
Post-RP PSA (Persistent vs. Non-Detectable)	<0.001	30.963	19.634	48.828
Pathological GLEASON Score	0.003	1.245	1.079	1.436
Pathological T Stage (T2 vs. T3–4)	<0.001	1.649	1.299	2.094
Surgical Margin Status (R0 vs. R1)	0.025	1.491	1.052	2.114
Pathological (p)N0 (Referent)	0.007			
Molecular İmaging (mi)N0pN1	0.481	1.160	0.768	1.752
miN1pN1	0.002	2.105	1.304	3.399
*Pre-surgery setting*				
PSA at Diagnosis	0.015	1.006	1.001	1.010
Biopsy Gleason Score	0.001	1.317	1.118	1.551
cT Stage	<0.001	1.282	1.152	1.427
miN Stage	<0.001	2.176	1.496	3.165

## Data Availability

Data supporting the findings of the study are available from the corresponding author [O.Ö.] on request.
